# Sensitivity of Metrics of Phylogenetic Structure to Scale, Source of Data and Species Pool of Hummingbird Assemblages along Elevational Gradients

**DOI:** 10.1371/journal.pone.0035472

**Published:** 2012-04-27

**Authors:** Sebastián González-Caro, Juan L. Parra, Catherine H. Graham, Jimmy A. McGuire, Carlos Daniel Cadena

**Affiliations:** 1 Laboratorio de Biología Evolutiva de Vertebrados, Departamento de Ciencias Biológicas, Universidad de los Andes, Bogotá, Colombia; 2 Instituto de Biología, Facultad de Ciencias Exactas y Naturales, Universidad de Antioquia, Medellín, Colombia; 3 Department of Ecology and Evolution, Stony Brook University, Stony Brook, New York, United States of America; 4 Department of Integrative Biology, University of California, Berkeley, California, United States of America; University of Copenhagen, Denmark

## Abstract

Patterns of phylogenetic structure of assemblages are increasingly used to gain insight into the ecological and evolutionary processes involved in the assembly of co-occurring species. Metrics of phylogenetic structure can be sensitive to scaling issues and data availability. Here we empirically assess the sensitivity of four metrics of phylogenetic structure of assemblages to changes in (i) the source of data, (ii) the spatial grain at which assemblages are defined, and (iii) the definition of species pools using hummingbird (Trochilidae) assemblages along an elevational gradient in Colombia. We also discuss some of the implications in terms of the potential mechanisms driving these patterns. To explore how source of data influence phylogenetic structure we defined assemblages using three sources of data: field inventories, museum specimens, and range maps. Assemblages were defined at two spatial grains: coarse-grained (elevational bands of 800-m width) and fine-grained (1-km^2^ plots). We used three different species pools: all species contained in assemblages, all species within half-degree quadrats, and all species either above or below 2000 m elevation. Metrics considering phylogenetic relationships among all species within assemblages showed phylogenetic clustering at high elevations and phylogenetic evenness in the lowlands, whereas those metrics considering only the closest co-occurring relatives showed the opposite trend. This result suggests that using multiple metrics of phylogenetic structure should provide greater insight into the mechanisms shaping assemblage structure. The source and spatial grain of data had important influences on estimates of both richness and phylogenetic structure. Metrics considering the co-occurrence of close relatives were particularly sensitive to changes in the spatial grain. Assemblages based on range maps included more species and showed less phylogenetic structure than assemblages based on museum or field inventories. Coarse-grained assemblages included more distantly related species and thus showed a more even phylogenetic structure than fine-grained assemblages. Our results emphasize the importance of carefully selecting the scale, source of data and metric used in analysis of the phylogenetic structure of assemblages.

## Introduction

Patterns of phylogenetic structure are increasingly being used to gain insight into the ecological and evolutionary processes involved in the assembly of co-occurring species [1]–[5]. However, metrics used to quantify phylogenetic structure among co-occurring species, and hence the conclusions drawn from such metrics, can be influenced by a number of factors. These factors include the regional species pool considered in testing for patterns of phylogenetic structure, the spatial grain at which assemblages are defined, and the source of data used to assess species composition of assemblages [1]–[2], [4], [6]–[7]. To date, studies have generally evaluated the individual influence of these factors, but they can also act in concert. Here, we explore how the species pool, spatial grain, and source of data, alone and in combination, influence the assessment of patterns of phylogenetic structure of hummingbird assemblages along an elevational gradient in the Colombian Andes.

The interpretation of indices of phylogenetic structure depends on how the species pool is defined [7]–[10] because phylogenetic structure is calculated by comparing pair-wise distances among co-occurring species to distances between pairs of species selected randomly from the pool. The two extreme outcomes are assemblages composed of either closer or more distant relatives than expected under a random assembly process (phylogenetic clustering or evenness, respectively). As the number of species in a pool increases, for example owing to an increase in taxonomic coverage, it is more likely to include more distant relatives. Thus, with larger species pools one expects a higher number of assemblages showing significant phylogenetic clustering and a lower number of assemblages showing significant phylogenetic evenness [9]. The degree of ecological realism involved in the definition of the species pool also has a marked influence on the inferences of mechanisms involved in assembly processes that can be made based on analyses of phylogenetic structure [8].

The spatial grain used to define an assemblage can also influence the magnitude, and thus, the interpretation of indices of phylogenetic structure [9]–[11]. On one hand, if the grain over which resource partitioning occurs approximates the scale at which assemblages are defined, then one expects biotic interactions, such as competition [12], predation [13] and mutualism [14] to play an important role in structuring assemblages. For example, if individuals of interacting species of hummingbirds have home ranges of ∼1 km^2^, these species could exclude each other at that scale if they compete for finite resources available in the area (e.g. flowers of a particular species). On the other hand, if the grain at which assemblages are defined approximates the scale over which there are spatial shifts in habitat types, then one expects assemblages to be structured by habitat filtering such that co-occurring species exhibit similar adaptations to those habitats, resulting in phylogenetic clustering. Further, at larger scales, evolutionary processes (speciation, extinction, and colonization) can be important drivers of phylogenetic structure [3]. Therefore, the combined effects of habitat filtering and biogeographic/evolutionary history may result in phylogenetic clustering as the spatial grain at which assemblages are defined increases [2], [7], [11], provided ecologically relevant traits are evolutionarily conserved. Nonetheless, patterns of phylogenetic structure across assemblages can be used to generate hypotheses about potential mechanisms structuring assemblages [2].

The source of data used to estimate assemblage composition can influence perceived spatial patterns of species composition and richness, and may also influence analyses of phylogenetic structure, but this has not been evaluated. Data from field inventories are commonly used to define assemblages for analyses of phylogenetic structure [15] because of their high spatial resolution and standardized sampling methods. However, the spatial distribution of field inventories may be restricted and therefore can underestimate species co-occurrence patterns at regional scales [15]. For example, field inventories are often performed in areas of easy access, potentially underestimating true species richness at regional scales. Other types of information commonly used in macroecological studies are museum records and species' range maps [5], [16]. Museum records have some of the same drawbacks as field inventories because sampling can be spatially biased (e.g., focused in areas with higher accessibility) [17] or may be biased by the technique used to collect specimens. Assemblage composition assessed from species range maps might overestimate species occurrence at fine spatial grains because it ignores the patchiness in the internal structure of species' distributions [18]. Therefore, relative to field inventories or museum records, range maps might be less able to detect signatures of species interactions occurring locally (e.g. competitive exclusion leading to phylogenetic evenness) [19]–[20].

Hummingbirds represent an useful system to study the effect of spatial grain, species pool and source of data on indices of measuring the phylogenetic structure of assemblages because their distributions are relatively well-known, which allows gathering data from different sources and at various spatial grains. Further, hummingbird ecology is well studied, which allows one to propose ecological explanations for variation in phylogenetic structure metrics among sources of data, spatial grains, and species pools. Hummingbirds likely originated in lowland Amazonia [21]–[22], but they are most diverse at intermediate elevations in the Andes [21]. At high elevations, flight is energetically costly and requires physiological and morphological adaptations [23]. Because only a few hummingbird clades have evolved such adaptations, phylogenetic clustering in the highlands (i.e., >2500 m elevation) supports the idea that habitat filtering influences assemblage composition at high elevations, whereas significant phylogenetic evenness in the lowlands is suggestive of competition [24]–[25].

Here we combine data on hummingbird assemblage composition across an elevational gradient in the Colombian Andes with a robust molecular phylogeny [22] to evaluate how the spatial grain at which assemblages are defined, the species pool considered in testing for patterns of phylogenetic structure, and the source of data used to assess species composition of assemblages influence metrics of phylogenetic structure. We also evaluate how do metrics of phylogenetic structure vary along the elevational gradient using four of the most commonly used indices of phylogenetic structure, the net relatedness and nearest taxon indices [26], and the phylogenetic species variability and the phylogenetic species clustering indices [27].

## Materials and Methods

### Study Area

**Figure 1:**
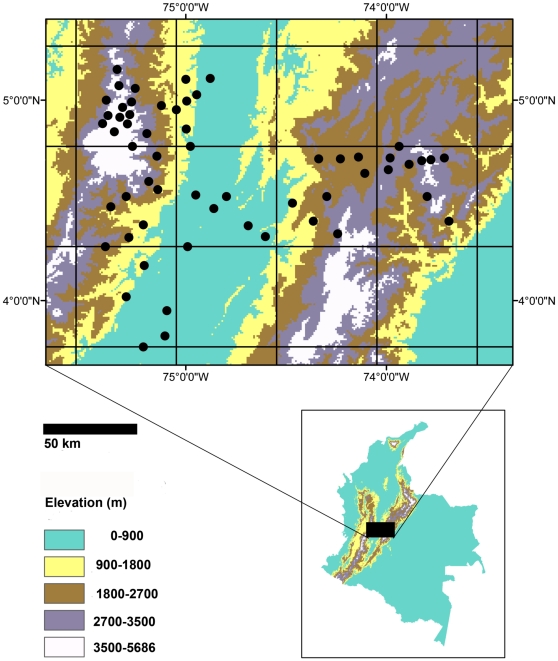
Geographic location of hummingbird assemblages. Geographic location of field inventories in the study area. Grids represent the half-degree grains used to define species pools. Color-scale elevation bands are classified according to the elevation categories used in analyses. Cities are indicated with triangles (Bogotá on the east and Manizales on the west).

We used data obtained along a topographically heterogeneous strip running west to east from the city of Manizales in the Cordillera Central of the Colombian Andes to the city of Bogotá, in the Cordillera Oriental (Fig. 1; the elevational range covered with our data was 250 to 4000 m). We chose this study region because it contains elevational gradients and has ample information on hummingbird distribution. The study area included high elevations on the eastern slope of the Cordillera Central and western slope of the Cordillera Oriental and the low-lying intervening Magdalena Valley.

### Phylogenetic Reconstruction

All 74 species of hummingbirds occurring in the study area according to range maps were included in a molecular phylogeny of 170 species. Phylogenetic relationships were estimated using DNA sequences from three nuclear genes: adenylate kinase intron 5 (AK1), beta fibrinogen intron 7 (Bfib), and ornythin decarboxylase intron 6 (ODC), and two mitochondrial genes: NADH dehydrogenase subunit 2 and 4 (ND2, ND4), comprising 4906 aligned base pairs. The phylogeny was estimated using a Bayesian method (MrBayes v. 3.1) [28] with separate partitions applied to each nuclear gene, and to each codon position within the mitochondrial genes and their flanking tRNAs (12 total partitions). Appropriate substitution models for each partition were determined using the Akaike Information Criterion (AIC) as implemented in the program ModelTest 3.06 [29]. The resulting tree is well resolved and supported, with 79% of nodes receiving posterior probabilities of 95% or greater. Most sequences had been included in previous phylogenetic analyses [22], [24] and are deposited in GenBank; new sequences for seven species added in this study have also been archived (all accession nos. in Table S1).

### Phylogenetic Structure

We calculated four indices of phylogenetic structure of assemblages: net relatedness index (NRI), nearest taxon index (NTI), phylogenetic species variability (PSV), and phylogenetic species clustering (PSC) [2], [27]. NRI is based on the mean pairwise phylogenetic distance (MPD) between all possible pairs of species in an assemblage. NTI uses the mean minimum phylogenetic distance (MMPD) calculated as the mean phylogenetic distance to the closest co-occurring relative. The difference between the observed and expected MPD and MMPD is standardized by the standard deviation of the distribution of null assemblages to represent the standardized effect size of each metric. We calculated significance of NRI and NTI for each assemblage by comparing the observed indices with the mean of 1000 assemblages simulated under the independent swap null model [27], [30]–[31]. This method avoids the possible inference of phylogenetic structure due to phylogenetic signal in species prevalence and as such is preferable relative to other null models [32]. Positives values of NRI and NTI indicate phylogenetic clustering and negative values indicate phylogenetic evenness [26]. The statistical significance of the phylogenetic structure of a group of assemblages was calculated using one sample t-tests where the null expectation is zero, i.e., a random sample of species with respect to phylogeny.

PSV and PSC measure the degree to which co-occurring species are related by comparing the expected variance of a hypothetical neutral trait evolving under Brownian motion along the phylogeny of the co-occurring species relative to the variance expected under a star phylogeny of the same species (i.e., a phylogeny representing a burst of radiation where all species evolve simultaneously from the same common ancestor and are thus equally distant from each other) [27]. Instead of using the full variance-covariance matrix, PSC only considers the portion including the closest relatives, so it can be thought of as analogous to NTI [29]. Values close to one indicate phylogenetic evenness whereas values close to zero indicate phylogenetic clustering [27]. The statistical significance of PSV and PSC was calculated using the mean of these indices across multiple assemblages contrasted against the null mean value of 1000 random assemblages [27]. PSV is mathematically related to NRI, with the main difference between them being the way they are standardized [27]. The relationship between PSC and NTI has not been studied [23].

Assemblages were grouped relative to assemblages defined at greater grain sizes (i.e., 1 km^2^ or grouped by elevational bands). NRI and PSV measure overall relatedness among all species in an assemblage whereas NTI and PSC measure phylogenetic distance among closest relatives within an assemblage. NRI and PSV are inversely related but the relationship between NTI and PSC has not been explored [27]. All indices were calculated using the package Picante v. 1.1-1 [33] for R v. 2.10.1 [34].

### Species Pool

We defined our species pools in three different ways. First, we used all 74 species present in all sources of data (inventories, museum specimens and range maps) and for which we had phylogenetic information. This is a practical definition of species pool without much ecological or biogeographical meaning. We defined a second species pool based on the species co-occurring in the half-degree quadrats, and a third one using two partitions: species occurring either above or below 2000 m elevation. The second species pool is defined based on a smaller area relative to the first definition of species pool and has no identified ecological or biogeographic meaning, but reflects a change in the spatial extent at which the pool is considered. The elevation threshold used to define the third species pool was chosen based on marked variation in assemblage composition documented in previous studies; specifically, hummingbirds in the hermit subfamily (Phaethorninae) are diverse and abundant at low elevations, but they largely drop out at c. 2000 m likely owing to functional constraints, such that high-elevation assemblages are composed entirely of nonhermits (Trochilinae) [35]. This last definition is expected to reflect more ecological or functional realism. We use the term species pool phylogeny to refer to the phylogeny based on each of the different species pools.

### Spatial Grain

We evaluated how indices of the phylogenetic structure of assemblages were influenced by grain size (coarse- and fine-grained) used to define assemblages. For the coarse spatial grain, we used the following elevation bands within half-degree quadrats: 250–900 m, 900–1800 m, 1800–2700 m, 2700–3500 m and 3500–5686 m. We chose these elevations as cutoffs between bands because biotic (i.e. vegetation) and abiotic conditions change significantly at these points along the gradient [36]. We acknowledge that all bands do not cover the same area (range 12 to 20 km^2^), but all bands covered the same elevational range, another potential factor influencing the composition of species in an area. The fine spatial grain was defined as 1-km^2^ quadrats distributed evenly across the study area as a grid. Areas of this size have been suggested to be the minimum spatial extents at which local avian assemblages are properly assessed in Neotropical forests [37].

### Sources of Data

Our analyses involved three sources of data commonly employed in studies of phylogenetic structure of assemblages: field inventories, museum specimen records and species' range maps [16]. Field inventories were lists of species from specific localities available from published literature (Text S1). We obtained data for a total of 59 field inventories spread relatively evenly across the study area (Fig. 1). Inventories generally sampled an area of *ca.* 1 km^2^. Assemblages assessed from museum records were based on occurrences for individual species included in the BIOMAP database, which contains Colombian specimen information collected between 1910 and 2000 (7000 species occurrence points) from 42 museums worldwide (http://www.biomap.net). We carefully checked the georeference for each locality and georeferenced additional specimens using the Instituto Geográfico Agustín Codazzi database (http://www.igac.gov.co). Finally, we estimated assemblage composition based on range maps developed by NatureServe (http://www.natureserve.org).

To scale field inventories up to the coarse spatial grain, we tallied all species across all field inventories for each elevational band. For museum specimens and range-map data, we determined all species occurring within 1-km^2^ pixels. For the coarse-grained assemblages, we intersected the different sources of species data sets with polygons representing each of the spatial grains to obtain assemblage composition using the program ArcGIS v. 9.3. This procedure resulted in 59 fine- and 21 coarse-grained assemblages for each source of data.

Because analyses of phylogenetic structure can be influenced by differences in species richness and composition, we quantified species richness for each of the three sources of data and spatial grains, and evaluated changes in species composition in relation to changes in source of data and spatial grain for each assemblage. To assess changes in assemblage composition as estimated by different sources of data, we calculated the compositional nestedness index as *C/min (A, B)*, where *C* is the number of species shared between both target assemblages and the denominator is the minimum species richness in any of the target assemblages *A* and *B* [38]–[39]. In our case, target assemblages refer to the same assemblage but generated with different sources of data (e.g., range maps versus museum records). The index equals 1 when all the species in the assemblage with lower species richness are represented in the assemblage with higher species richness; a value of zero indicates that assemblages do not share species.

### Statistical Analyses

Prior to conducting statistical tests, we checked for assumptions of normality and homogeneity of variances. First, we compared changes in assemblage composition among different sources of data using a nested analysis of variance (ANOVA), where each assemblage was treated as a subject and source of data was a nested factor. Second, to compare the effect of species pool on metrics of phylogenetic structure we used a nested ANOVA, where each assemblage was treated as a subject and species pool was a nested factor. Finally, we evaluated the sensitivity of estimates of richness and phylogenetic structure to spatial grain and source of data using a two-way analysis of covariance (ANCOVA). Elevation was used as the covariate (i.e., the mean elevation of a given assemblage at a given spatial grain). This analysis allowed us to test for differences between spatial grains among data sets used while correcting for the effect of elevation. When comparing among grain sizes, the statistical power of the ANCOVA can be compromised because the number of assemblages differs at each grain size (fine-grain, n = 59; coarse-grain, n = 21). Thus, in addition to an ANCOVA using all data, we used a rarefaction analysis [10] where we randomly sampled (without replacement) a subset of the fine-grained assemblages and used the same sample size for both grain sizes in a corrected ANCOVA. All analyses were performed using packages vegan v. 1.17 and base in R.

## Results

### Effects of Species Pool, Spatial Grain, and Source of Data on Patterns of Species Richness

**Figure 2:**
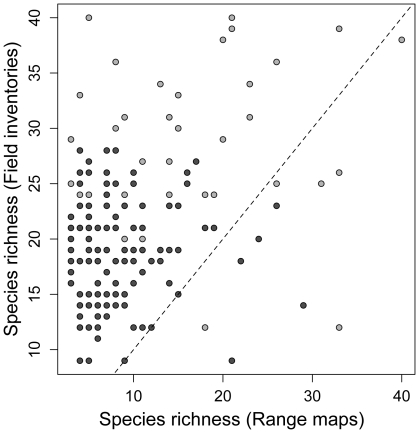
Species richness obtained from different sources at different scales. Relationship between species richness estimated from field inventories and range maps at two different spatial grains. Coarse-grained assemblages are shown in light gray and fine-grained assemblages in dark gray. The line indicates one-to-one correspondence, demonstrating that richness estimated from range maps are higher than those estimated from field inventories.

Elevation had a significant effect on species richness (Table 1). Species richness and its standard deviation were higher at intermediate elevations (mean±sd = 17.75±10.41, between 1800 and 2700 m) relative to high (mean±sd = 16.60±10.60, between 2700 and 4000 m) and low (mean±sd = 13±9.40, between 250 and 1800 m) elevations (Fig. S1). Species richness also varied significantly with respect to source of data (Table 1) and spatial grain (Table 1). There was no significant interaction between source of data and spatial grain (Table 1). Assemblage richness estimated from range maps was higher than that based on field inventories and museum records (Tables 1 & 2, Fig. 2). Estimates of richness were higher at the coarse spatial grain than at the fine spatial grain (Tables 1 & 2, Fig. 2).

**Table 1:** Results of the ANCOVA testing for differences in species richness and in the four indices of phylogenetic structure when changing spatial grains and sources of data using elevation as a covariable.

	df	SS	MS	*F*	*P*	Tukey HSD
*Species Richness*						
Spatial grain	1	2965.20	2965.20	85.45	**0.00**	
Data source	2	8761.70	4380.90	126.25	**0.00**	All comparisons
Elevation	1	429.70	429.70	12.38	**0.00**	
Grain * Data	2	2.30	1.20	0.03	0.97	
*NRI*						
Spatial grain	1	3.97	3.97	1.92	0.17	
Data source	2	3.25	1.63	0.79	0.46	
Elevation	1	316.62	316.62	153.47	**0.00**	
Grain * Data	2	9.65	4.83	2.34	0.10	
*NTI*						
Spatial grain	1	6.69	6.69	6.06	**0.01**	Coarse-grain<Fine-grain
Data source	2	5.69	2.84	2.58	0.08	
Elevation	1	7.89	7.89	7.15	**0.01**	
Grain * Data	2	0.75	0.37	0.34	0.71	
*PSV*						
Spatial grain	1	0.01	0.01	1.70	0.19	
Data source	2	0.28	0.14	25.35	**0.00**	Maps>Museum; Maps>Field
Elevation	1	0.55	0.55	98.98	**0.00**	
Grain * Data	2	0.01	0.00	0.49	0.62	
*PSC*						
Spatial grain	1	0.03	0.03	4.14	**0.04**	Coarse-grain>Fine-grain
Data source	2	0.46	0.23	28.32	**0.00**	Maps>Museum; Maps>Field
Elevation	1	0.20	0.20	24.07	**0.00**	
Grain * Data	2	0.00	0.00	0.20	0.82	

Significant *P*-values are highlighted in bold. Only significant Tukey post-hoc comparisons are mentioned.

**Table 2:** Mean and standard deviation of richness and of the four indices of phylogenetic structure for each combination of spatial grain and source of data used.

Spatial grain	Data type	Richness	NRI			NTI			PSV			PSC		
		Mean (SD)	Mean (SD)	Even (%)	Clustered (%)	Mean (SD)	Even (%)	Clustered (%)	Mean (SD)	Even (%)	Clustered (%)	Mean (SD)	Even (%)	Clustered (%)
***Coarse grain***	**Field inventories**	27.7 (8.70)	0.75 (1.42)	14.3	28.6	−0.36 (1.20)	28.6	14.3	**0.82** (0.04)	0	71.4	**0.54** (0.03)	71.4	0
	**Museum records**	31.3 (11.5)	**1.11** (1.09)	28.6	28.6	0.34 (1.12)	14.3	14.3	**0.82** (0.02)	0	71.4	**0.58** (0.07)	61.6	0
	**Range maps**	36.8 (2.50)	−0.54 (0.68)	14.3	14.3	−**0.62** (0.68)	0	14.3	**0.85** (0.01)	0	0	**0.58** **(0.03)**	71.4	0
***Fine grain***	**Field inventories**	8.40 (4.53)	**0.71** (1.29)	11.8	28.8	0.21 (0.95)	28.6	14.3	**0.81** (0.07)	0	20.3	**0.39** (0.11)	20.3	0
	**Museum records**	7.80 (5.12)	**1.07** (1.28)	12.6	27.9	**0.86** (1.12)	14.3	14.3	**0.78** (0.09)	0	30.4	**0.45** (0.12)	30.4	0
	**Range maps**	19.5 (4.90)	−0.22 (1.14)	30.1	37	0.08 (1.01)	0	13.4	**0.85** (0.03)	0	80.8	**0.52** (0.08)	61.1	0

Percentages of assemblages showing statistically significant phylogenetic evenness or clustering are indicated.

The species nestedness composition index was equal to 1 in almost all cases (field inventories vs. museum including both spatial grains: median = 1, range = 0.99–1; field inventories vs. range maps: median = 1, range = 0.97–1; museum records vs. range maps: median = 1, range = 0.97–1; ANOVA, *F* = 0.10; *P*>0.99). These results indicate that assemblages with fewer species are subsets of assemblages with more species.

### Effects of Species Pool, Spatial Grain, and Source of Data on Patterns of Phylogenetic Structure

We did not find differences in indices of phylogenetic structure calculated based on any of the species pools (i.e. there was no effect of species pool on assessments of phylogenetic structure; NRI, *F* = 1.03, *P*>0.05; NTI, *F* = 0.08, *P*>0.05; PSV, *F* = 0.1, *P*>0.50; PSC, *F* = 0.54, *P*>0.05). There were also no differences in the relationship (i.e., slope) between indices of phylogenetic structure and elevation when applying different definitions of the species pool (Table S2). The distribution of assemblages with significant patterns of phylogenetic structure was also very similar across species pools (Table S3). Therefore, in subsequent analyses we only describe results based on the total species pool including all 74 species.

**Figure 3:**
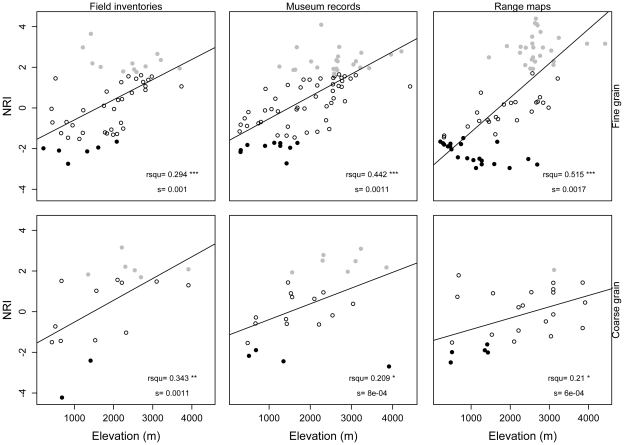
Relationship between the Net Relatedness Index (NRI) and elevation. Relationship between NRI and elevation. Assemblages showing statistically significant patterns of phylogenetic evenness are represented with black filled circles, those with phylogenetic clustering in gray circles, and those showing patterns not deviating from the null model in hollow circles. R-squared values (rsqu) and the slope (s) of the linear regression are shown on the lower right corner (* = p<0.05, ** = p<0.01, *** = p<0.001).

**Figure 4:**
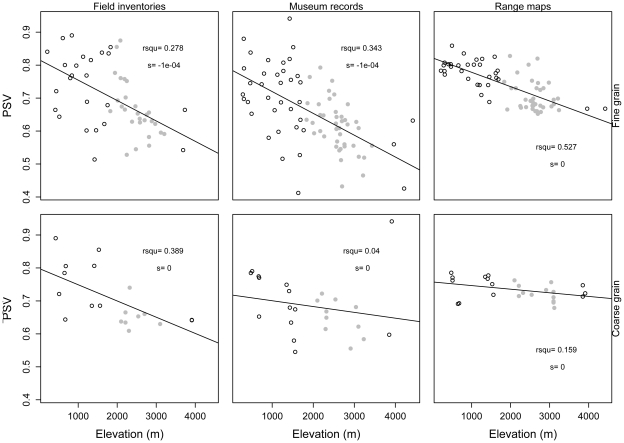
Relationship between the Phylogenetic Species Variability index (PSV) and elevation. Relationship between PSV and elevation. Assemblages showing statistically significant patterns of phylogenetic evenness are represented with black filled circles, those with phylogenetic clustering in gray circles, and those showing patterns not deviating from the null model in hollow circles. R-squared values (rsqu) and the slope (s) of the linear regression are shown on the lower right corner (* = p<0.05, ** = p<0.01, *** = p<0.001).

**Figure 5:**
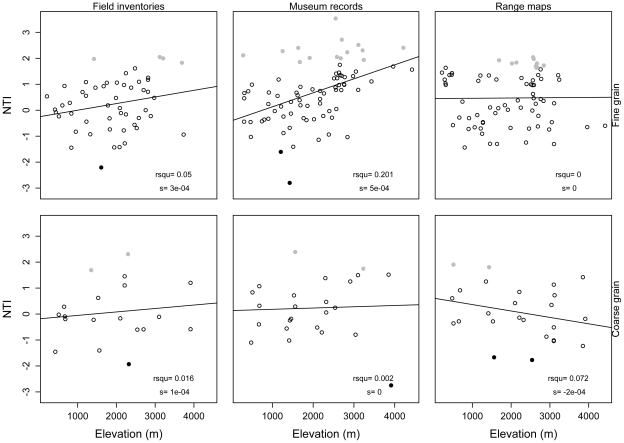
Relationship between the Nearest Taxon Index (NTI) and elevation. Relationship between NTI and elevation. Assemblages showing statistically significant patterns of phylogenetic evenness are represented with black filled circles, those with phylogenetic clustering in gray circles, and those showing patterns not deviating from the null model in hollow circles. R-squared values (rsqu) and the slope (s) of the linear regression are shown on the lower right corner (* = p<0.05, ** = p<0.01, *** = p<0.001).

**Figure 6:**
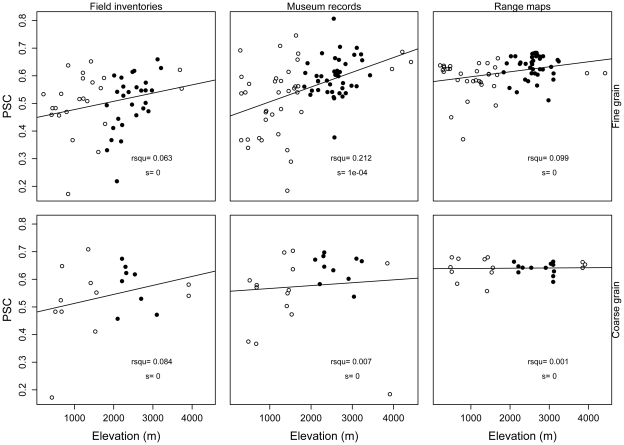
Relationship between the Phylogenetic Species Clustering index (PSC) and elevation. Relationship between PSC and elevation. Assemblages showing statistically significant patterns of phylogenetic evenness are represented with black filled circles, those with phylogenetic clustering in gray circles, and those showing patterns not deviating from the null model in hollow circles. R-squared values (rsqu) and the slope (s) of the linear regression are shown on the lower right corner (* = p<0.05, ** = p<0.01, *** = p<0.001).

All four indices of phylogenetic structure varied with elevation (ANCOVA, Table 1; Fig. S2). Results of NRI and PSV (which are negatively related) were similar, with phylogenetic evenness at low elevations and clustering at high elevations (Figs. 3, 4). NTI showed the weakest pattern with respect to elevation of the four indices, and its pattern varied depending on spatial grain and source of data (Fig. 5). PSC showed the opposite pattern to NRI and PSV; according to PSC lowland assemblages were more phylogenetically clustered than highland assemblages (Fig. 6).

There were significant differences among sources of data used to estimate community composition in PSV and PSC (Tables 1 & S4), but not in NRI or NTI (Tables 1 & S4). Values of PSV and PSC for assemblages derived from field inventories and museum records were similar and were more clustered than the same indices calculated for assemblages based on range maps (Tables 1, 2 & S4). Nonetheless, the interpretation of the raw averages of PSV and PSC among data sources is similar (Table 2). For example, the mean phylogenetic structure according to PSV is always clustered regardless of data source, whereas the mean phylogenetic structure according to PSC is always even regardless of data source.

The effect of the spatial grain on patterns of phylogenetic structure varied across indices (Table 1). NRI and PSV showed similar results across spatial grains (Tables 1 & S4), whereas NTI and PSC were different between fine and coarse spatial grains. At coarse spatial grains, both NTI and PSC changed significantly toward relatively even values (i.e., values of NTI decreased while values of PSC increased). These differences are reflected in the slopes of the relationships between elevation and each index across spatial grains (Figs. 3, 4, 5, and 6). The same results were found when differences in sample size between spatial grains were controlled for using rarefaction (Table S5).

## Discussion

A variety of methodological and conceptual decisions influence quantification of the phylogenetic structure of assemblages and the inferences drawn from these analyses. Despite the known effects of changes in the species pool on metrics of phylogenetic structure [4]–[6], [8], [10], [40], we found that these metrics are robust to some changes in the species pool. However, we found that the source of data used to estimate assemblage composition had a significant effect on measures of phylogenetic structure, which may make comparisons among studies difficult. Our results suggest that in areas with good sampling, local assemblage composition can be approximated using museum specimen records, but that range-map data overestimate composition at fine spatial grains. The spatial grain at which hummingbird assemblages were defined in our study affected only metrics indicative of relatedness among closest relatives (PSC and NTI), but not measures of overall relatedness (NRI and PSV) of co-occurring species.

In general, phylogenetically clustered hummingbird assemblages occurred in the highlands, possibly as a consequence of habitat filtering, whereas lowland assemblages tended to be phylogenetically even, presumably due to competitive interactions [24]–[25] or to the evolutionary origin of several lineages of hummingbirds in the wet lowlands [21], [41]. However, PSC revealed an inverse trend (i.e., evenness in the highlands). This discrepancy is noteworthy but difficult to interpret. It remains possible that the recovery of inverse patterns by different indices reflects that, in addition to environmental filtering, competition among close relatives might be shaping patterns of phylogenetic structure at high elevations. Our results show that PSV and PSC tended to be more sensitive to changes in the composition of assemblages (either because of a change in spatial grain or a change in the source of data used to establish the composition of assemblages) than NRI and NTI. We believe this is a desirable property of an index of phylogenetic structure. Nevertheless, choosing among indices of phylogenetic structure is ultimately dependent on the research question [27] and other information available (see below). In the remainder of the discussion, we first address the differences among the indices of phylogenetic structure used in this study and then we focus on the effects of species pool, source of data, spatial grain, and elevation on our estimates of the phylogenetic structure of hummingbird assemblages.

### Metrics of Phylogenetic Structure

The four metrics of phylogenetic structure used in this study are intended to measure either different aspects of the evolutionary relationships among co-occurring species or are based on different methodologies. Our results are consistent with previous work in that NRI and PSV show similar patterns [27]. In contrast, NTI and PSC did not covary across the elevational gradient or spatial grains (see below). The main differences between these metrics is that NTI is standardized by the mean and standard deviation of nearest-neighbor distances sampled randomly from the species pool phylogeny, whereas PSC is standardized only relative to a star phylogeny including all species in the pool. Thus, although NTI is sensitive to both changes in assemblage composition and in the mean expectation from the species pool, PSC is only sensitive to changes in assemblage composition. Nonetheless, the mathematical relationship between these two metrics has not been studied in detail [27]. Our results suggest that these metrics can respond differently to changes in assemblage composition, with PSV and PSC being more sensitive (i.e., being able to detect more subtle differences in phylogenetic composition) than NRI or NTI to changes in the composition of assemblages. Previous work on these metrics [27] recommends the use of PSV and PSC because they are not affected by species richness and abundance. We agree with [27] and in addition, we believe that another advantage of PSV and PSC is that they are standardized relative to a star phylogeny. A point of reference is needed to interpret indices of phylogenetic structure. NRI and NTI use the mean value of randomly assembled assemblages under a specified null model as a point of reference. This point of reference is affected by particularities of the species pool phylogeny like its shape [40]. Thus, the magnitude and sign of NRI and NTI are affected by the topology of the species pool phylogeny. In contrast, PSV and PSC use as a point of reference the expected variance of a hypothetical trait evolving under Brownian motion under a situation when all members of an assemblage are equally related (star phylogeny) [27]. This point of reference is independent of the topology of the species pool phylogeny. Thus, at least in terms of comparing phylogenetic structure indices across studies, it might be more reasonable to use indices such as PSV and PSC, which use a theoretical situation (a star phylogeny) as a reference point rather than an empirical situation (an observed tree topology). Note that we are referring to values of PSV and PSC values, the statistical significance of which is evaluated in relation to randomly defined assemblages. Ultimately, future research is required to evaluate when and why inferences obtained from these indices might vary.

### Species Pool

Although several studies have shown that the size of the species pool influences indices of phylogenetic structure [6]–[8], [41], we saw no effect of the change in species pool on any of the indices considered in our study. This may be a result of the extent of our study area, or of the way in which we designated the species pool relative to the elevational gradient. The extent of our study area can be considered relatively small for birds, and thus, partitions within this extent may not affect the distribution of phylogenetic distances in the species pool if all major clades are included in all species pools. For example, our pools based on elevation (below and above 2000 m), while excluding either highland or lowland specialists, included the majority of species found at middle elevations. In addition, although hermits (Phaethorninae) as a whole decline markedly in diversity with elevation, one species belonging to this clade (*Phaethornis syrmatophorus*) does reach areas above 2000 m. As a result, both types of pools included species from across the entire regional phylogeny. Therefore, we observed minimal differences among metrics calculated using different pools [5], highlighting the robustness of these metrics to slight changes in the species pool [6]. Nonetheless, our results should not be interpreted as to indicate that species-pool designation has generally no influence on metrics of phylogenetic structure. We only suggest that these metrics should remain robust to changes in the composition of the species pool that do not greatly affect the distribution of phylogenetic distances. A recent paper [8] emphasizes the importance of defining species pools following explicit ecological criteria rather than on the basis of spatial extents. We included species pools defined under both criteria and did not find significant differences in patterns of phylogenetic structure.

### Spatial Grain

As expected from the species-area relationship, we found that species richness is greatest when assemblages are defined at the largest spatial grain [42]–[43]. This change in assemblage size can affect the statistical power of metrics of phylogenetic structure [7]. This is evident in the change of the percentage of assemblages showing significant patterns of phylogenetic evenness or clustering with the change in spatial grain (see Table 2, Table S5, Fig. S3). In addition, the species added to the assemblage at increasing spatial grains can affect the pattern of phylogenetic structure [44]–[45]. We found no change in mean phylogenetic structure across grain sizes for either NRI or PSV. This means that, on average, similar phylogenetic patterns were detected when the spatial grain at which the assemblage was defined increased. This is not consistent with the idea that the species added to the assemblage when one increases the spatial grain are random draws from the phylogeny, but rather suggests they include species that reinforce patterns of phylogenetic structure found at fine spatial grains. In contrast, there were differences between fine and coarse spatial grains in indices that measure phylogenetic structure based on the closest relatives (NTI and PSC). The change in these indices from fine to coarse spatial grains is indicative of an increase of phylogenetic evenness with the species added to assemblages. This trend is particularly apparent in assemblages at low elevations and likely reflects the geography of our study region, where low elevations are confined to a narrow river valley. Thus, when increasing the spatial grain at low elevations, taxa from distant clades (e.g., the two Andean clades: brilliants and coquettes) are included, whereas at high elevations mostly members of the same clades are included. This result is consistent with the idea that the highest phylogenetic turnover occurs at the interface between lowlands and mountain slopes [24].

### Source of Data

Assemblage richness estimated from field inventories and museum records was similar and lower than richness estimated from range maps. This result is consistent with our expectation; local assemblages assessed from range maps have higher richness because range maps lack detail on local species-environment associations and species interactions, which can lead to overestimation of the number of species co-occurring locally [15]. Further, assuming that field inventories represent relatively complete lists of species at a fine spatial grain, then our result suggests that museum records can be useful for assessing patterns of species composition at fine spatial grains [46]. Of course, this need not be true in cases where collection activity has been low, and especially when collectors may favor (or avoid) species belonging to particular clades. Like richness, the phylogenetic structure of assemblages was also influenced by source of data, though this result was sensitive to the index used. Values of PSV and PSC for assemblages derived from field inventories and museum records were similar, highlighting the potential usefulness of museum data despite the fact that specimens are often not collected in a systematic way with the goal of producing complete and unbiased local inventories. However, when calculated using range maps, these indices tended to show no phylogenetic structure (i.e., more similar to null model expectation). When assemblages contain a larger portion of the species represented in the entire species pool, as is the case with range maps, it is more difficult to identify differences in species composition in a given assemblage and the total pool using null models [7]. In contrast to the values of PSV and PSC, values of NRI and NTI were similar across all sources of data.

More broadly, our results indicate that the source of data can have an important influence on estimates of both richness and phylogenetic structure; to date, this had only been acknowledged for species richness [15], [47]. A variety of sources for presence/absence data have been used to calculate the phylogenetic structure of assemblages including range maps [48]–[49] and inventories conducted at a range of spatial extents (i.e., plot-level and transect-based samples). Range maps may provide adequate broad-scale assessments of structure in large regions but may be less appropriate for evaluating species co-occurrence patterns at small scales [15], [47].

### Phylogenetic Structure Along the Elevational Gradient

NTI, NRI and PSV showed increased phylogenetic clustering with elevation. This result likely reflects the extensive diversification of the two hummingbird clades at high elevations in the Andes [22], [50]. In addition, this result is consistent with the habitat-filtering hypothesis suggested by Graham *et al*. [24]–[25], where closely related species co-occur at high elevations due to their presumably shared tolerance for highland conditions (e.g. cold temperatures, low oxygen pressure, reduced air density) [50]. However, measurements of the evolutionary lability of ecological traits are needed to test this assumption [28]. In contrast, PSC showed an opposite pattern of phylogenetic structure along the elevational gradient; highland assemblages tended to be phylogenetically even, especially when defined at the fine spatial grain. Because PSC is a measure of phylogenetic distance among closest relatives, and because competition is thought to be strongest between close relatives, this index may more easily detect the influence of biotic interactions on the phylogenetic structure of an assemblage [2], [7], [27]. Combined, these results indicate that at high elevations competition for resources might influence patterns of species co-occurrence (especially at fine spatial grains), at the same time that the conditions varying with elevation may act as a habitat filter. Several studies have used divergent patterns of phylogenetic structure to hypothesize that a mixture of ecological processes, including habitat filtering and competition, influence the structure of assemblages [46], [51]. Finally, in the lowlands, phylogenetic evenness was apparent at both spatial grains in NRI, NTI and PSV, and evenness was most pronounced at the coarse spatial grain.

In sum, we conducted a sensitivity analysis of four commonly used metrics of phylogenetic structure to changes in the spatial grain at which assemblages are defined, the species pool used, and sources of data. Our results indicate that measures of phylogenetic structure involving only the closest co-occurring relatives are more sensitive to changes in spatial grain than metrics that include all co-occurring species. This is partly due to the smaller number of pairwise relations used to estimate phylogenetic structure when only closest relatives are considered and is also dependent on the geographic context of the assemblage. Assemblages assessed based on field inventories and museum specimens provide different estimates than those based on range maps, which usually contain more species. As found in previous studies focusing on other regions [24], phylogenetic structure of hummingbird assemblages varies greatly along the elevational gradient. At high elevations, hummingbird assemblages tend to be phylogenetically clustered relative to assemblages at low elevations, especially in humid forests. In addition to this trend, we found that when only including close co-occurring relatives, there is evidence of evenness at high elevations relative to assemblages in the lowlands. These multiple patterns of phylogenetic structure suggest that both biotic interactions and environmental filtering may influence patterns of assemblage composition [25].

## Supporting Information

Text S1 List of references for field inventories.
(DOC)

Figure S1 Relationship between species richness and elevation for all combinations of spatial grains and sources of data.
(TIF)

Figure S2 Distribution of major hummingbird clades along the elevational gradient based on field inventories and museum records in this study.
(TIF)

Figure S3 Proportion of assemblages with significant patterns of phylogenetic structure at different spatial grains and based on different sources of data. Dark gray indicates coarse-grained assemblages and light gray indicates fine-grained assemblages. Differences between combinations based on *Chi*-square tests are indicated (* = *P*<0.05, ** = *P*<0.01, *** = *P*<0.001).
(TIF)

Table S1 GenBank accession numbers for all sequences of species used to estimate the overall phylogeny (ALL, 170 species) that included the 74 species present in the assemblages (ASS). Sequences with no data are labeled as ‘no seqs’ and sequences still pending accession numbers as ‘pending’.
(DOCX)

Table S2 Estimated slope values and their respective 95% confidence intervals from the relationship between phylogenetic structure and elevation for each phylogenetic structure index (NRI, NTI, PSV, PSC) under each unique combination of species pool, spatial grain and data source.
(DOC)

Table S3 Percentage of assemblages with significant patterns of phylogenetic structure (even or clustered) for each combination of spatial grain, data source and species pool.
(DOC)

Table S4 Results of *Tukey* post-hoc comparisons of *ANCOVA* analyses among sources of data and spatial grains (*t*-value (*P*-value)). Significant *P*-values are highlighted in bold (RM = Range maps, FI = Field inventories, MR = Museum records, CO = Coarse-grained assemblages, FN = Fine-grained assemblages).
(DOC)

Table S5 Results of the ANCOVA analyses using the same number of samples per spatial grain. Significant *P*-values are highlighted in bold.
(DOC)
