# Supplementary material for: Sensitivity of Metrics of Phylogenetic Structure to Scale, Source of Data and Species Pool of Hummingbird Assemblages along Elevational Gradients
Source: PLoS One. 2012 Apr 27;7(4):e35472. doi: 10.1371/journal.pone.0035472 (PMC3338702; doi:10.1371/journal.pone.0035472)
Supplement: Text S1 — List of references for field inventories. (DOC) [file pone.0035472.s001.doc]

**Supplementary Material.**  González et al.

**Text S1.** References of studies from which lists of hummingbird species were obtained for analyses based on field inventories.

Duque, D. 2004. Listado de las aves del Quindío (1988-2004). Corporación Autónoma Regional del Quindío. Unpublished.

Echeverry-Galvis, M.A. & Morales-Rozo, A. (2007). Lista anotada de algunas especies de la vereda “Cerca de Piedra”, Chía, Colombia. *Boletín SAO (Sociedad Antioqueña de Ornitologia)*,**17**, 87-93

Gutiérrez-Zamora, A. (2008).Las interacciones ecológicas y estructura de una comunidad altoandina de colibríes y flores en la cordillera oriental de Colombia. *Ornitología Colombiana*,**7**,17-42

Losada-Prado, S., Carvajal-Lozano, A., &. Molina-Martínez, G. (2005). Listado de especies de aves de la cuenca del río Coello (Tolima, Colombia). *Biota Colombiana,* **6**,101-116

Moreno-Salazar, N. & Pedro Camargo-Martínez, P. (2008). Inventario preliminar de la avifauna de la vereda “Acuapal” municipio de Sasaima – Cundinamarca. *Boletín SAO (Sociedad Antioqueña de Ornitologia)*,**18**, 15.

Moreno-Salazar, N. & Camargo-Martínez, P. (2008). Registros de las aves de cuatro zonas del Parque Nacional Natural Chingaza. *Boletín SAO (Sociedad Antioqueña de Ornitologia)*,**18**, 14.

Naranjo, S.L. & Abril, B.C. (1995). Inventario y aspectos ecológicos de la avifauna existente en el relicto vegetal de la Universidad del Quindío, Armenia, Quindío. *Biología y Educación*, **5**,19-30.

Parra-Hernández, R.M., Carantón-Ayala, D., Sanabria-Mejía J., Barrera-Rodríguez, F., Sierra-Sierra, A., Moreno-Palacios, M., Yate-Molina, W., Figueroa-Martínez, W., Díaz-Jaramillo, C., Florez-Delgado, V., Certuche-Cubillos, K., Loaiza-Hernández, H. & Florido-Cuellar, B. (2007). Aves del municipio de Ibagué - Tolima, Colombia. *Biota Colombiana*, **8**,199-220.

Stiles, F.G. & Roselli, L. (1998). Inventario de las aves de un bosque altoandino: comparación de dos métodos. *Caldasia*,**20**, 29-43

Verhelst, J.C., Rodríguez, J., Orrego, O., Botero, J., López, J., Franco V., & Pfeifer A. (2001). Aves del Municipio de Manizales- Caldas, Colombia. *Biota Colombiana*, **3**, 265-284.
