# Supplementary figures and images for: Sensitivity of Metrics of Phylogenetic Structure to Scale, Source of Data and Species Pool of Hummingbird Assemblages along Elevational Gradients
Source: PLoS One. 2012 Apr 27;7(4):e35472. doi: 10.1371/journal.pone.0035472 (PMC3338702; doi:10.1371/journal.pone.0035472)

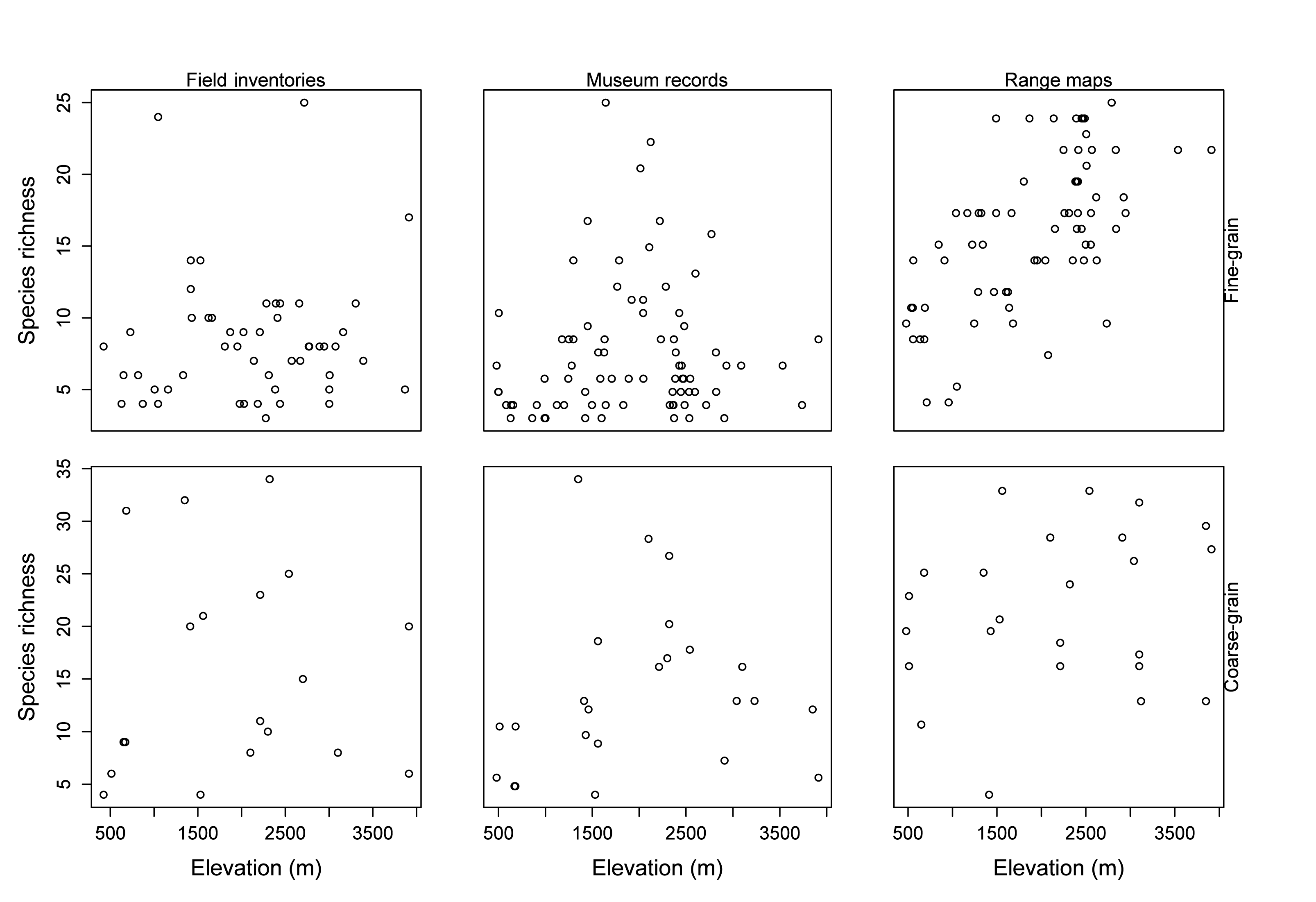

Supplement: Figure S1 — Relationship between species richness and elevation for all combinations of spatial grains and sources of data. (TIF) [file pone.0035472.s002.tif]

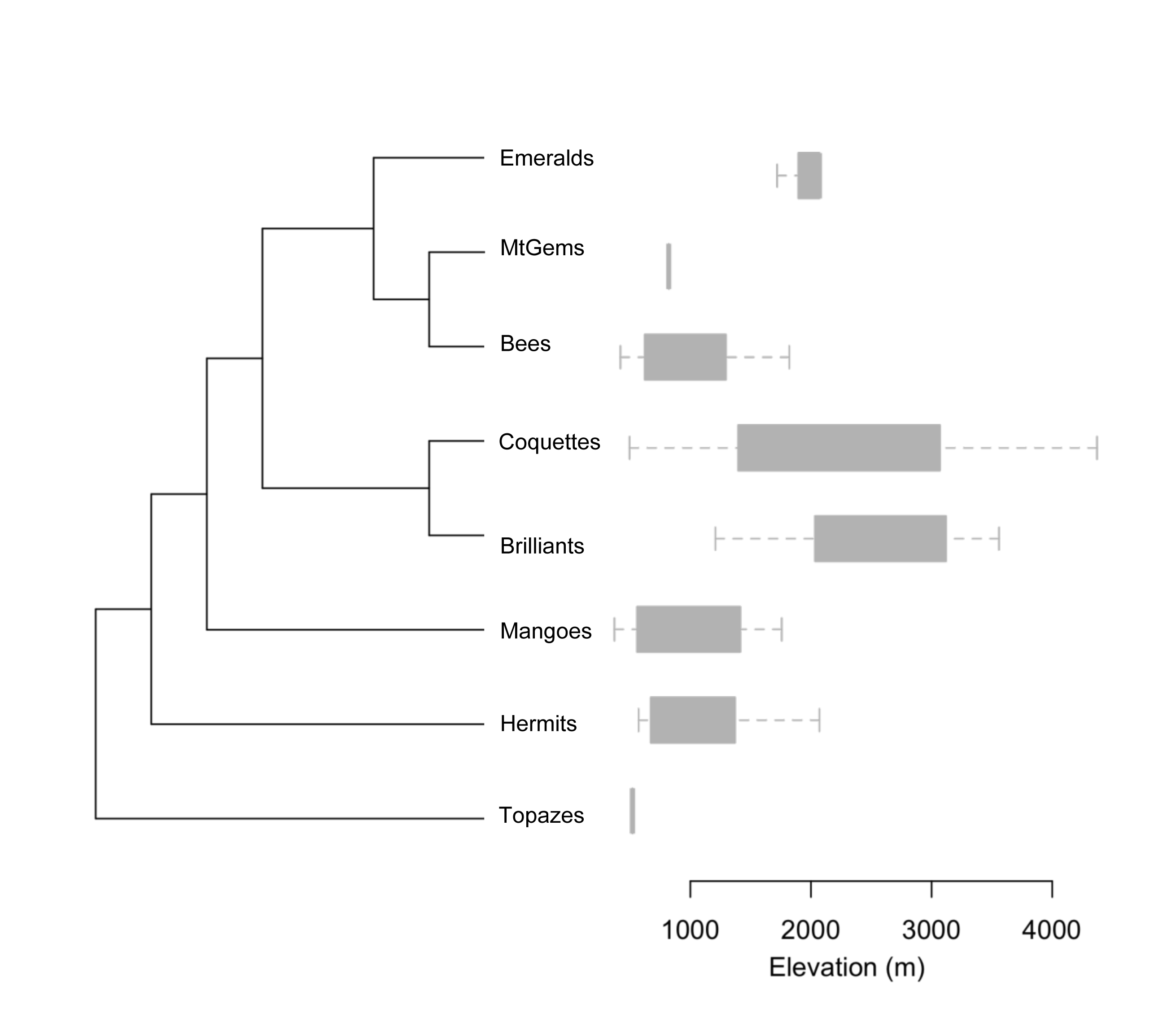

Supplement: Figure S2 — Distribution of major hummingbird clades along the elevational gradient based on field inventories and museum records in this study. (TIF) [file pone.0035472.s003.tif]

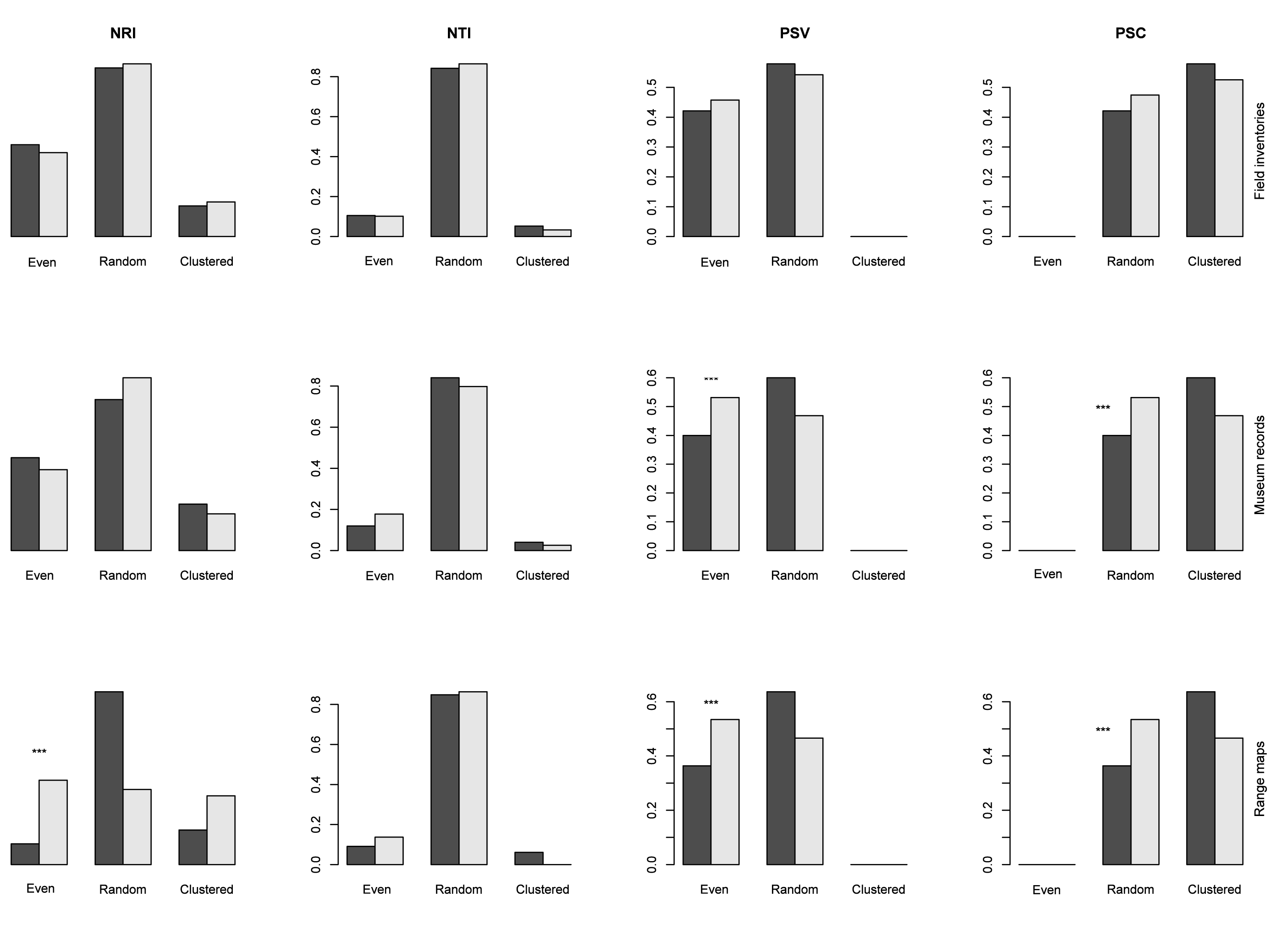

Supplement: Figure S3 — Proportion of assemblages with significant patterns of phylogenetic structure at different spatial grains and based on different sources of data. Dark gray indicates coarse-grained assemblages and light gray indicates fine-grained assemblages. Differences between combinations based on Chi-square tests are indicated (* = P<0.05, ** = P<0.01, *** = P<0.001). (TIF) [file pone.0035472.s004.tif]
