# Supplementary material for: Sensitivity of Metrics of Phylogenetic Structure to Scale, Source of Data and Species Pool of Hummingbird Assemblages along Elevational Gradients
Source: PLoS One. 2012 Apr 27;7(4):e35472. doi: 10.1371/journal.pone.0035472 (PMC3338702; doi:10.1371/journal.pone.0035472)
Supplement: Table S1 — GenBank accession numbers for all sequences of species used to estimate the overall phylogeny (ALL, 170 species) that included the 74 species present in the assemblages (ASS). Sequences with no data are labeled as ‘no seqs’ and sequences still pending accession numbers as ‘pending’. (DOCX) [file pone.0035472.s005.docx]

**Table S1**. GenBank accession numbers for all sequences of species used to estimate the overall phylogeny (ALL, 170 species) that included the 74 species present in the assemblages (ASS). Sequences with no data are labeled as ‘no seqs’ and sequences still pending accession numbers as ‘pending’.

| **SPECIES** | **ND2** | **ND4** | **BFIB** | **AK1** | **ODC** | **ALL** | **ASS** |
| --- | --- | --- | --- | --- | --- | --- | --- |
| *Adelomyia melanogenys* | AY830457 | EU042274 | AY830608 | AY830533 | GU166937 | x | x |
| *Aglaeactis cupripennis* | AY830459 | EU042276 | FJ903573 | FJ903645 | FJ903537 | x | x |
| *Aglaeactis pamela* | GU167201 | GU166869 | GU167091 | GU167147 | GU166939 | x |  |
| *Aglaiocercus coelestis* | AY830460 | EU042277 | AY830611 | AY830536 | GU166940 | x | x |
| *Agliocercus kingi* | AY830461 | EU042278 | AY830612 | AY830537 | GU166941 | x | x |
| *Amazilia amabilis* | EU042518 | EU042198 | EU042354 | EU042436 | GU166942 | x | x |
| *Amazilia amazilia* | GU167202 | GU166870 | GU167092 | GU167148 | GU166943 | x |  |
| *Amazilia castaneiventris* | PENDING | PENDING | PENDING | PENDING | PENDING | x |  |
| *Amazilia chionogaster* | AY830462 | EU042279 | AY830613 | AY830538 | GU166944 | x |  |
| *Amazilia fimbriata* | EU042520 | EU042200 | EU042356 | EU042438 | GU166945 | x | x |
| *Amazilia franciae* | EU042521 | EU042201 | EU042357 | EU042439 | GU166946 | x | x |
| *Amazilia lactea* | GU167203 | GU166871 | GU167093 | GU167149 | GU166947 | x |  |
| *Amazilia rosenbergi* | GU167204 | GU166872 | GU167094 | GU167150 | GU166948 | x |  |
| *Amazilia saucerrottei* | GU167205 | GU166873 | GU167095 | GU167151 | GU166949 | x | x |
| *Amazilia tzacatl* | EU042524 | EU042204 | EU042360 | EU042442 | GU166950 | x | x |
| *Amazilia versicolor* | EU042525 | EU042205 | EU042361 | EU042443 | GU166951 | x | x |
| *Amazilia viridicauda* | GU167206 | GU166874 | GU167096 | GU167152 | GU166952 | x |  |
| *Amazilia viridigaster* | GU167207 | GU166875 | GU167097 | GU167153 | GU166953 | x | x |
| *Androdon aequatorialis* | AY830463 | EU042280 | AY830614 | AY830539 | GU166954 | x |  |
| *Anthocephala floriceps* | GU167208 | GU166876 | GU167098 | GU167154 | GU166955 | x | x |
| *Anthracothorax nigricollis* | EU042527 | EU042207 | EU042363 | EU042445 | GU166956 | x |  |
| *Anthracothorax prevostii* | GU167209 | GU166877 | GU167099 | GU167155 | GU166957 | x |  |
| *Avocettula recurvirostris* | GU167210 | GU166878 | GU167100 | GU167156 | GU166958 | x |  |
| *Boissoneaua mathewsii* | AY830466 | EU042283 | AY830617 | AY830542 | GU166960 | x |  |
| *Boissonneaua flavescens* | EU042530 | EU042210 | EU042366 | EU042448 | GU166959 | x | x |
| *Calliphlox amethystina* | GU167211 | GU166879 | GU167101 | GU167157 | GU166961 | x |  |
| *Calliphlox mitchellii* | AY830516 | GU166880 | AY830667 | AY830592 | GU166962 | x |  |
| *Campylopterus falcatus* | GU167212 | GU166881 | GU167102 | GU167158 | GU166963 | x | x |
| *Campylopterus largipennis* | AY830467 | EU042285 | AY830618 | AY830543 | GU166964 | x |  |
| *Campylopterus villaviscensio* | AY830468 | EU042286 | AY830619 | AY830544 | GU166965 | x |  |
| *Chaetocercus bombus* | GU167213 | GU166882 | GU167103 | GU167159 | GU166966 | x |  |
| *Chaetocercus mulsant* | AY830456 | EU042287 | AY830607 | AY830532 | GU166967 | x | x |
| *Chalcostigma herrani* | EU042536 | EU042216 | EU042372 | EU042454 | GU166968 | x | x |
| *Chalcostigma ruficeps* | AY830469 | EU042288 | AY830620 | AY830545 | GU166969 | x |  |
| *Chalcostigma stanleyi* | GU167255 | GU166924 | GU167144 | no seqs | GU167090 | x |  |
| *Chalybura buffonii* | EU042537 | EU042217 | EU042373 | EU042455 | GU166970 | x | x |
| *Chalybura urochrysia* | EU042538 | EU042218 | EU042374 | EU042456 | GU166971 | x |  |
| *Chlorestes notata* | EU042539 | EU042219 | EU042375 | EU042457 | GU166972 | x |  |
| *Chlorostilbon aureoventris* | GU167214 | GU166883 | GU167104 | GU167160 | GU166973 | x |  |
| *Chlorostilbon melanorhynchus* | AY830470 | EU042289 | AY830621 | AY830546 | GU166974 | x |  |
| *Chlorostilbon mellisugus* | AY830471 | EU042290 | AY830622 | AY830547 | GU166975 | x | x |
| *Chlorostilbon notatus* | EU042539 | EU042219 | EU042375 | EU042457 | GU166972 | x |  |
| *Chlorostilbon poortmani* | GU167215 | GU166884 | GU167105 | GU167161 | GU166976 | x |  |
| *Chrysolampis mosquitus* | EU042540 | EU042220 | EU042376 | EU042458 | GU166977 | x | x |
| *Chrysuronia oenone* | AY830472 | EU042291 | AY830623 | AY830548 | GU166978 | x |  |
| *Coeligena bonapartei* | FJ903502 | FJ903610 | FJ903574 | FJ903646 | FJ903538 | x | x |
| *Coeligena coeligena* | FJ960666 | FJ960719 | FJ960621 | FJ960569 | FJ960762 | x | x |
| *Coeligena helianthea* | FJ903510 | FJ903618 | FJ903582 | FJ903654 | FJ903546 | x | x |
| *Coeligena iris* | GU167218 | GU166887 | GU167108 | GU167164 | GU166979 | x |  |
| *Coeligena lutetiae* | EU042542 | EU042222 | FJ903589 | FJ903661 | FJ903553 | x | x |
| *Coeligena orina* | GU167219 | GU166888 | GU167109 | GU167165 | GU167109 | x |  |
| *Coeligena phalerata* | FJ903521 | FJ903629 | FJ903593 | FJ903665 | FJ903557 | x |  |
| *Coeligena prunellei* | GU167221 | GU166890 | GU167111 | GU167167 | PENDING | x |  |
| *Coeligena torquata* | AY830473 | EU042292 | AY830624 | AY830549 | GU166980 | x | x |
| *Coeligena violifer* | AY830474 | EU042293 | AY830625 | AY830550 | GU166981 | x |  |
| *Coeligena wilsoni* | AY830475 | EU042294 | AY830626 | AY830551 | GU166982 | x |  |
| *Colibri coruscans* | AY830476 | EU042295 | AY830627 | GU166829 | GU166983 | x | x |
| *Colibri delphinae* | EU042543 | EU042223 | EU042379 | EU042461 | GU166984 | x | x |
| *Colibri serrirostris* | GU167222 | GU166891 | GU167112 | GU167168 | GU166985 | x | x |
| *Colibri thalassinus* | EU042544 | EU042224 | EU042380 | EU042462 | GU166986 | x |  |
| *Damophila julie* | EU042545 | EU042225 | EU042381 | EU042463 | GU166987 | x | x |
| *Discosura langsdorffi* | GU167223 | GU166892 | GU167113 | GU167169 | GU166989 | x |  |
| *Discosura longicauda* | GU167224 | GU166893 | GU167114 | GU167170 | GU166990 | x |  |
| *Discosura popelairii* | EU042546 | EU042226 | EU042382 | EU042464 | GU166991 | x |  |
| *Doryfera johannae* | EU042547 | EU042227 | EU042383 | EU042465 | GU166992 | x | x |
| *Doryfera ludoviciae* | PENDING | EU042297 | PENDING | PENDING | GU166993 | x | x |
| *Ensifera ensifera* | AY830479 | EU042299 | FJ903606 | FJ903678 | FJ903570 | x | x |
| *Eriocnemis alinae* | EU042549 | EU042229 | EU042385 | EU042467 | GU166994 | x | x |
| *Eriocnemis cupreoventris* | GU167225 | GU166894 | GU167115 | GU167171 | GU166995 | x |  |
| *Eriocnemis derbyi* | PENDING | PENDING | no seqs | no seqs | no seqs | x | x |
| *Eriocnemis glaucopoides* | GU167226 | GU166895 | GU167116 | GU167172 | GU166996 | x |  |
| *Eriocnemis luciani* | AY830480 | EU042300 | AY830631 | AY830555 | GU166997 | x |  |
| *Eriocnemis mosquera* | EU042550 | EU042230 | EU042386 | PENDING | GU166998 | x | x |
| *Eriocnemis nigrivestis* | GU167227 | GU166896 | GU167117 | GU167173 | GU166999 | x |  |
| *Eriocnemis vestita* | EU042551 | EU042231 | EU042387 | GU166830 | GU167000 | x | x |
| *Eupetomena macroura* | GU167228 | GU166897 | GU167118 | GU167174 | GU167001 | x |  |
| *Eutoxeres aquila* | AY830483 | EU042304 | AY830634 | AY830558 | GU167002 | x | x |
| *Eutoxeres condamini* | AY830484 | EU042305 | AY830635 | AY830559 | GU167003 | x |  |
| *Florisuga fusca* | GU167229 | GU166898 | GU167119 | GU167175 | GU167004 | x |  |
| *Florisuga mellivora* | AY830485 | EU042306 | AY830636 | AY830560 | GU167005 | x | x |
| *Glaucis aeneus* | EU042554 | EU042234 | EU042390 | EU042470 | GU167006 | x |  |
| *Glaucis hirsutus* | AY830486 | EU042307 | AY830637 | AY830561 | GU167007 | x | x |
| *Haplophaedia aureliae* | AY830487 | EU042308 | AY830638 | AY830562 | GU167008 | x | x |
| *Haplophaedia lugens* | EU042555 | EU042235 | EU042391 | EU042471 | GU167009 | x |  |
| *Heliangelus amethysticollis* | AY830489 | EU042310 | AY830640 | AY830564 | GU167010 | x | x |
| *Heliangelus exortis* | EU042556 | EU042236 | EU042392 | EU042472 | GU167011 | x | x |
| *Heliangelus micraster* | GU167230 | GU166899 | GU167120 | GU167176 | GU167012 | x |  |
| *Heliangelus strophianus* | GU167231 | GU166900 | GU167121 | GU167177 | GU167013 | x |  |
| *Heliangelus viola* | GU167232 | GU166901 | GU167122 | GU167178 | GU167014 | x |  |
| *Heliodoxa aurescens* | AY830518 | EU042311 | AY830669 | AY830593 | GU167015 | x |  |
| *Heliodoxa branickii* | AY830490 | EU042312 | AY830641 | AY830566 | GU167016 | x |  |
| *Heliodoxa gularis* | GU167233 | GU166902 | GU167123 | GU167179 | GU167017 | x |  |
| *Heliodoxa imperatrix* | EU042557 | EU042237 | EU042393 | EU042473 | GU167018 | x |  |
| *Heliodoxa jacula* | AY830491 | EU042313 | AY830642 | AY830566 | GU167019 | x | x |
| *Heliodoxa leadbeateri* | AY830492 | EU042314 | AY830643 | AY830567 | GU167020 | x | x |
| *Heliodoxa rubinoides* | EU042558 | EU042238 | EU042394 | EU042474 | GU167021 | x | x |
| *Heliodoxa schreibersii* | EU042559 | EU042239 | EU042395 | EU042475 | GU167022 | x |  |
| *Heliomaster furcifer* | GU167234 | GU166903 | GU167124 | GU167180 | GU167023 | x |  |
| *Heliomaster longirostris* | AY830493 | EU042315 | AY830644 | AY830568 | GU167024 | x | x |
| *Heliothryx auritus* | GU167235 | GU166904 | GU167125 | GU167181 | GU167025 | x |  |
| *Heliothryx barroti* | AY830494 | EU042316 | AY830645 | AY830569 | GU167026 | x | x |
| *Hylocharis chrysura* | GU167236 | GU166905 | GU167126 | GU167182 | GU167027 | x |  |
| *Hylocharis cyanus* | EU042561 | EU042241 | EU042397 | EU042477 | GU167028 | x |  |
| *Hylocharis grayi* | EU042563 | EU042243 | no seqs | no seqs | no seqs | x | x |
| *Hylocharis sapphirina* | EU042564 | EU042244 | EU042399 | EU042479 | GU167029 | x |  |
| *Klais guimeti* | AY830495 | EU042317 | AY830646 | AY830571 | GU167030 | x |  |
| *Lafresnaya lafresnayi* | AY830496 | EU042318 | FJ903608 | FJ903680 | FJ903572 | x | x |
| *Lepidopyga caeruleogularis* | AY830497 | EU042319 | AY830648 | AY830572 | GU167031 | x |  |
| *Lepidopyga goudoti* | PENDING | PENDING | PENDING | PENDING | PENDING | x | x |
| *Lesbia nuna* | AY830498 | EU042320 | AY830649 | AY830573 | GU167032 | x | x |
| *Lesbia victoriae* | AY830499 | GU166862 | AY830650 | AY830574 | GU167033 | x | x |
| *Leucippus baeri* | GU167237 | GU166906 | GU167127 | GU167183 | GU167034 | x |  |
| *Leucippus chlorocercus* | GU167238 | GU166907 | GU167128 | GU167184 | GU167035 | x |  |
| *Lophornis chalybeus* | GU167239 | GU166908 | GU167129 | GU167185 | GU167036 | x |  |
| *Lophornis delattrei* | AY830500 | EU042321 | AY830651 | AY830575 | GU167037 | x | x |
| *Metallura aeneocauda* | AY830501 | EU042322 | AY830652 | AY830576 | GU167038 | x |  |
| *Metallura baroni* | GU167240 | GU166909 | GU167130 | GU167186 | GU167039 | x |  |
| *Metallura odomae* | GU167241 | GU166910 | GU167131 | GU167187 | GU167040 | x |  |
| *Metallura phoebe* | EU042569 | EU042249 | EU042404 | EU042484 | GU167041 | x |  |
| *Metallura tyrianthina* | AY830502 | EU042323 | AY830653 | AY830577 | GU167042 | x | x |
| *Metallura williami* | EU042570 | EU042250 | EU042405 | EU042485 | GU167043 | x |  |
| *Microstilbon burmeisteri* | GU167242 | GU166911 | GU167132 | GU167188 | GU167044 | x |  |
| *Myrmia micrura* | GU167243 | GU166912 | GU167133 | GU167189 | GU167045 | x |  |
| *Myrtis fanny* | AY830503 | GU166865 | AY830654 | AY830578 | GU167046 | x |  |
| *Ocreatus underwoodii* | AY830504 | EU042324 | AY830655 | AY830579 | GU167047 | x | x |
| *Opisthoprora euryptera* | EU042572 | EU042252 | EU042407 | EU042487 | GU167048 | x | x |
| *Oreotrochilus chimborazo* | AY830506 | EU042326 | AY830657 | AY830581 | GU167049 | x |  |
| *Oreotrochilus estella* | AY830507 | EU042327 | AY830658 | AY830582 | GU167050 | x |  |
| *Oxypogon guerinii* | EU042573 | no seqs | EU042408 | EU042488 | no seqs | x | x |
| *Patagona gigas* | AY830510 | EU042330 | AY830661 | AY830585 | GU167051 | x |  |
| *Phaethornis anthophilus* | PENDING | PENDING | PENDING | PENDING | GU167053 | x | x |
| *Phaethornis atrimentalis* | EU042575 | EU042254 | EU042410 | EU042490 | GU167054 | x |  |
| *Phaethornis augusti* | EU042576 | EU042255 | EU042411 | EU042491 | GU167055 | x | x |
| *Phaethornis bourcieri* | EU042577 | EU042256 | EU042412 | EU042492 | GU167056 | x |  |
| *Phaethornis eurynome* | GU167245 | GU166914 | GU167135 | GU167191 | GU167057 | x |  |
| *Phaethornis griseogularis* | EU042578 | EU042257 | EU042413 | EU042493 | GU167058 | x |  |
| *Phaethornis guy* | PENDING | EU042331 | PENDING | PENDING | GU167059 | x | x |
| *Phaethornis hispidus* | AY830512 | EU042332 | AY830663 | AY830587 | GU167060 | x | x |
| *Phaethornis koepckeae* | GU167246 | GU166915 | AY830664 | AY830589 | GU167061 | x |  |
| *Phaethornis longirostris* | EU042579 | EU042258 | EU042414 | EU042494 | GU167062 | x |  |
| *Phaethornis longuemareus* | EU042580 | EU042259 | EU042415 | EU042495 | GU167063 | x | x |
| *Phaethornis malaris* | AY830514 | EU042334 | AY830665 | AY830589 | GU167064 | x |  |
| *Phaethornis philippii* | EU042581 | EU042260 | EU042416 | EU042496 | GU167065 | x |  |
| *Phaethornis pretrei* | GU167247 | GU166916 | GU167136 | GU167192 | GU167066 | x |  |
| *Phaethornis ruber* | AY830515 | EU042335 | AY830666 | AY830590 | GU167067 | x |  |
| *Phaethornis subochraceus* | GU167248 | GU166917 | GU167137 | GU167193 | GU167068 | x |  |
| *Phaethornis superciliosus* | GU167254 | GU166923 | GU167143 | no seqs | GU167089 | x | x |
| *Phaethornis syrmatophorus* | EU042583 | EU042262 | EU042418 | EU042498 | GU167069 | x | x |
| *Phaethornis yaruqui* | EU042584 | EU042263 | EU042419 | EU042499 | GU167070 | x |  |
| *Phlogophilus hemileucurus* | AY830517 | EU042336 | AY830668 | AY830592 | GU167071 | x |  |
| *Polytmus guainumbi* | EU042585 | EU042264 | EU042420 | EU042500 | GU167072 | x |  |
| *Polytmus theresiae* | EU042586 | EU042265 | EU042421 | EU042501 | GU167073 | x |  |
| *Popelairia conversii* | AY830519 | EU042296 | AY830670 | AY830594 | GU166988 | x | x |
| *Pterophanes cyanopterus* | AY830520 | EU042337 | AY830671 | AY830595 | GU167074 | x | x |
| *Ramphomicron microrhynchum* | EU042587 | EU042266 | EU042422 | EU042502 | GU167075 | x | x |
| *Sappho sparganura* | GU167249 | GU166918 | GU167138 | GU167194 | GU167076 | x |  |
| *Schistes geoffroyi* | AY830521 | EU042338 | AY830672 | AY830596 | GU167077 | x | x |
| *Stephanoxis lalandi* | GU167250 | GU166919 | GU167139 | GU167195 | GU167078 | x |  |
| *Taphrospilus hypostictus* | AY830523 | EU042340 | AY830674 | AY830598 | GU167079 | x |  |
| *Thalurania colombica* | AY830524 | EU042342 | AY830675 | AY830600 | GU167080 | x | x |
| *Thalurania fannyi* | GU167257 | GU166926 | GU167146 | no seqs | no seqs | x |  |
| *Thalurania furcata* | AY830525 | EU042341 | AY830676 | AY830600 | GU167081 | x | x |
| *Thalurania glaucopis* | GU167251 | GU166920 | GU167140 | GU167196 | GU167082 | x |  |
| *Thaumastura cora* | GU167252 | GU166921 | GU167141 | GU167197 | GU167083 | x |  |
| *Threnetes leucurus* | AY830526 | EU042343 | AY830677 | AY830601 | GU167084 | x |  |
| *Threnetes ruckeri* | AY830527 | EU042344 | AY830678 | AY830602 | GU167085 | x | x |
| *Topaza pella* | AY830528 | EU042345 | AY830679 | AY830603 | PENDING | x |  |
| *Urochroa bougueri* | EU042594 | EU042273 | EU042429 | EU042509 | GU167087 | x | x |
| *Urosticte benjamani* | AY830529 | EU042346 | AY830680 | AY830604 | GU167088 | x | x |
| *Urosticte ruficrissa* | GU167256 | GU166925 | GU167145 | GU167199 | no seqs | x | x |
