# Supplementary material for: Sensitivity of Metrics of Phylogenetic Structure to Scale, Source of Data and Species Pool of Hummingbird Assemblages along Elevational Gradients
Source: PLoS One. 2012 Apr 27;7(4):e35472. doi: 10.1371/journal.pone.0035472 (PMC3338702; doi:10.1371/journal.pone.0035472)
Supplement: Table S2 — Estimated slope values and their respective 95% confidence intervals from the relationship between phylogenetic structure and elevation for each phylogenetic structure index (NRI, NTI, PSV, PSC) under each unique combination of species pool, spatial grain and data source. (DOC) [file pone.0035472.s006.doc]

**Table S2**. Estimated slope values and their respective 95% confidence intervals from the relationship between phylogenetic structure and elevation for each phylogenetic structure index (NRI, NTI, PSV, PSC) under each unique combination of species pool, spatial grain and data source.

| **Species pool** | **Spatial grain** | **Data source** | **NRI vs Elevation slope** | |
| --- | --- | --- | --- | --- |
|  |  |  | ***2.5*** | ***97.5*** |
| All species | Fine | Field | 5.4E-04 | 1.4E-03 |
|  |  | Musem | 3.0E-04 | 1.8E-03 |
|  |  | Maps | -5.2E-05 | 4.6E-03 |
|  | Coarse | Field | 8.2E-04 | 1.4E-03 |
|  |  | Musem | 9.3E-05 | 1.4E-03 |
|  |  | Maps | -3.6E-04 | 4.4E-03 |
| Elevational partitions | Fine | Field | 5.4E-04 | 1.4E-03 |
|  |  | Musem | 3.0E-04 | 1.8E-03 |
|  |  | Maps | -5.2E-05 | 4.6E-03 |
|  | Coarse | Field | 8.2E-04 | 1.4E-03 |
|  |  | Musem | 9.3E-05 | 1.4E-03 |
|  |  | Maps | -3.6E-04 | 4.4E-03 |
| Half-degree quadrat | Fine | Field | 5.4E-04 | 1.4E-03 |
|  |  | Musem | 3.0E-04 | 1.8E-03 |
|  |  | Maps | -5.2E-05 | 4.6E-03 |
|  | Coarse | Field | 8.2E-04 | 1.4E-03 |
|  |  | Musem | 9.3E-05 | 1.4E-03 |
|  |  | Maps | -3.6E-04 | 4.4E-03 |
| **Species pool** | **Spatial grain** | **Data source** | **NTI vs Elevation slope** | |
|  |  |  | ***2.5*** | ***97.5*** |
| All species | Fine | Field | -6.0E-05 | 5.7E-04 |
|  |  | Musem | -4.0E-04 | 6.6E-04 |
|  |  | Maps | -4.0E-03 | 3.1E-03 |
|  | Coarse | Field | 2.9E-04 | 7.8E-04 |
|  |  | Musem | -4.3E-04 | 5.6E-04 |
|  |  | Maps | -1.2E-03 | 2.6E-03 |
| Elevational partitions | Fine | Field | -6.0E-05 | 5.7E-04 |
|  |  | Musem | -4.0E-04 | 6.6E-04 |
|  |  | Maps | -4.0E-03 | 3.1E-03 |
|  | Coarse | Field | 2.9E-04 | 7.8E-04 |
|  |  | Musem | -4.3E-04 | 5.6E-04 |
|  |  | Maps | -1.2E-03 | 2.6E-03 |
| Half-degree quadrat | Fine | Field | -6.0E-05 | 5.7E-04 |
|  |  | Musem | -4.0E-04 | 6.6E-04 |
|  |  | Maps | -4.0E-03 | 3.1E-03 |
|  | Coarse | Field | 2.9E-04 | 7.8E-04 |
|  |  | Musem | -4.3E-04 | 5.6E-04 |
|  |  | Maps | -1.2E-03 | 2.6E-03 |
| **Species pool** | **Spatial grain** | **Data source** | **PSV vs Elevation slope** | |
|  |  |  | ***2.5*** | ***97.5*** |
| All species | Fine | Field | -9.0E-05 | -3.3E-05 |
|  |  | Musem | -8.1E-05 | -1.8E-05 |
|  |  | Maps | -1.4E-04 | 7.2E-06 |
|  | Coarse | Field | -8.7E-05 | -4.5E-05 |
|  |  | Musem | -5.6E-05 | 2.1E-05 |
|  |  | Maps | -7.9E-05 | 9.1E-06 |
| Elevational partitions | Fine | Field | -9.0E-05 | -3.3E-05 |
|  |  | Musem | -8.1E-05 | -1.8E-05 |
|  |  | Maps | -1.4E-04 | 7.2E-06 |
|  | Coarse | Field | -8.7E-05 | -4.5E-05 |
|  |  | Musem | -5.6E-05 | 2.1E-05 |
|  |  | Maps | -7.9E-05 | 9.1E-06 |
| Half-degree quadrat | Fine | Field | -9.0E-05 | -3.3E-05 |
|  |  | Musem | -8.1E-05 | -1.8E-05 |
|  |  | Maps | -1.4E-04 | 7.2E-06 |
|  | Coarse | Field | -8.7E-05 | -4.5E-05 |
|  |  | Musem | -5.6E-05 | 2.1E-05 |
|  |  | Maps | -7.9E-05 | 9.1E-06 |
| **Species pool** | **Spatial grain** | **Data source** | **PSC vs Elevation slope** | |
|  |  |  | ***2.5*** | ***97.5*** |
| All species | Fine | Field | -3.3E-06 | 6.3E-05 |
|  |  | Musem | -2.2E-05 | 8.7E-05 |
|  |  | Maps | -7.5E-05 | 5.3E-05 |
|  | Coarse | Field | 3.0E-05 | 7.7E-05 |
|  |  | Musem | -4.3E-05 | 6.4E-05 |
|  |  | Maps | -7.6E-06 | 1.3E-04 |
| Elevational partitions | Fine | Field | -3.3E-06 | 6.3E-05 |
|  |  | Musem | -2.2E-05 | 8.7E-05 |
|  |  | Maps | -7.5E-05 | 5.3E-05 |
|  | Coarse | Field | 3.0E-05 | 7.7E-05 |
|  |  | Musem | -4.3E-05 | 6.4E-05 |
|  |  | Maps | -7.6E-06 | 1.3E-04 |
| Half-degree quadrat | Fine | Field | -3.3E-06 | 6.3E-05 |
|  |  | Musem | -2.2E-05 | 8.7E-05 |
|  |  | Maps | -7.5E-05 | 5.3E-05 |
|  | Coarse | Field | 3.0E-05 | 7.7E-05 |
|  |  | Musem | -4.3E-05 | 6.4E-05 |
|  |  | Maps | -7.6E-06 | 1.3E-04 |
