# Supplementary material for: Sensitivity of Metrics of Phylogenetic Structure to Scale, Source of Data and Species Pool of Hummingbird Assemblages along Elevational Gradients
Source: PLoS One. 2012 Apr 27;7(4):e35472. doi: 10.1371/journal.pone.0035472 (PMC3338702; doi:10.1371/journal.pone.0035472)
Supplement: Table S3 — Percentage of assemblages with significant patterns of phylogenetic structure (even or clustered) for each combination of spatial grain, data source and species pool. (DOC) [file pone.0035472.s007.doc]

**Table S3**. Percentage of assemblages with significant patterns of phylogenetic structure (even or clustered) for each combination of spatial grain, data source and species pool.

| All species | Source of data and spatial grain | NRI | | NTI | | PSV | | PSC | |
| --- | --- | --- | --- | --- | --- | --- | --- | --- | --- |
|  |  | even | clustered | even | clustered | even | clustered | even | clustered |
|  | *Coarse-grain* | (%) | (%) | (%) | (%) | (%) | (%) | (%) | (%) |
|  | Field inventories | 10.5 | 31.6 | 5.7 | 10.5 | 0 | 42.1 | 0 | 0 |
|  | Museum records | 16 | 32 | 4 | 12 | 0 | 32 | 0 | 0 |
|  | Range maps | 15.1 | 9 | 6.1 | 9.1 | 0 | 0 | 0 | 0 |
|  | *Fine-grain* |  |  |  |  |  |  |  |  |
|  | Field inventories | 11.8 | 28.8 | 28.6 | 14.3 | 0 | 20.3 | 20.3 | 0 |
|  | Museum records | 12.6 | 27.9 | 14.3 | 14.3 | 0 | 30.4 | 30.4 | 0 |
|  | Range maps | 30.1 | 37 | 0 | 13.4 | 0 | 80.8 | 61.1 | 0 |
|  |  |  |  |  |  |  |  |  |  |
| Elevation | Type of data and spatial grain | NRI | | NTI | | PSV | | PSC | |
|  |  | even | clustered | even | clustered | even | clustered | even | clustered |
|  | *Coarse-grain* | (%) | (%) | (%) | (%) | (%) | (%) | (%) | (%) |
|  | Field inventories | 10.5 | 31.6 | 5.7 | 10.5 | 0 | 42.1 | 0 | 0 |
|  | Museum records | 16 | 32 | 4 | 12 | 0 | 32 | 0 | 0 |
|  | Range maps | 15.1 | 9 | 6.1 | 9.1 | 0 | 0 | 0 | 0 |
|  | *Fine-grain* |  |  |  |  |  |  |  |  |
|  | Field inventories | 11.8 | 25.4 | 28.6 | 17.6 | 0 | 20.3 | 20.3 | 0 |
|  | Museum records | 12.6 | 27.9 | 14.3 | 14.3 | 0 | 30.4 | 30.4 | 0 |
|  | Range maps | 30.1 | 39 | 0 | 9.56 | 0 | 80.8 | 61.1 | 0 |
|  |  |  |  |  |  |  |  |  |  |
| Half-degree | Type of data and spatial grain | NRI | | NTI | | PSV | | PSC | |
|  |  | even | clustered | even | clustered | even | clustered | even | clustered |
|  | *Coarse-grain* | (%) | (%) | (%) | (%) | (%) | (%) | (%) | (%) |
|  | Field inventories | 10.5 | 31.6 | 5.7 | 10.5 | 0 | 42.1 | 0 | 0 |
|  | Museum records | 16 | 32 | 4 | 12 | 0 | 32 | 0 | 0 |
|  | Range maps | 15.1 | 9 | 6.1 | 9.1 | 0 | 0 | 0 | 0 |
|  | *Fine-grain* |  |  |  |  |  |  |  |  |
|  | Field inventories | 13.7 | 28.8 | 28.6 | 14.3 | 0 | 20.3 | 20.3 | 0 |
|  | Museum records | 12.6 | 24.9 | 14.3 | 14.3 | 0 | 30.4 | 30.4 | 0 |
|  | Range maps | 30.1 | 35.2 | 0 | 13.4 | 0 | 80.8 | 61.1 | 0 |
|  |  |  |  |  |  |  |  |  |  |
