# Supplementary material for: Sensitivity of Metrics of Phylogenetic Structure to Scale, Source of Data and Species Pool of Hummingbird Assemblages along Elevational Gradients
Source: PLoS One. 2012 Apr 27;7(4):e35472. doi: 10.1371/journal.pone.0035472 (PMC3338702; doi:10.1371/journal.pone.0035472)
Supplement: Table S4 — Results of Tukey post-hoc comparisons of ANCOVA analyses among sources of data and spatial grains (t-value (P-value)). Significant P-values are highlighted in bold (RM = Range maps, FI = Field inventories, MR = Museum records, CO = Coarse-grained assemblages, FN = Fine-grained assemblages). (DOC) [file pone.0035472.s008.doc]

**Table S4.** Results of *Tukey* post-hoc comparisons of *ANCOVA* analyses among sources of data and spatial grains (*t*-value (*P*-value)). Significant *P*-values are highlighted in bold (RM=Range maps, FI=Field inventories, MR= Museum records, CO= Coarse-grained assemblages, FN=Fine-grained assemblages).

| **NRI** |  |  |  |  |
| --- | --- | --- | --- | --- |
|  | Difference | Lower | Upper | P-value |
| *Fine- vs Coarse-grained assemblages* | 0.17 | -0.30 | 0.64 | 0.490 |
| *Range maps-Field inventories* | -0.11 | -0.75 | 0.52 | 0.910 |
| *Museum records-Field inventories* | 0.10 | -0.54 | 0.73 | 0.930 |
| *Museum records-Range maps* | 0.21 | -0.37 | 0.79 | 0.680 |
| **NTI** |  |  |  |  |
| *Fine- vs Coarse-grained assemblages* | 0.23 | -0.05 | 0.52 | **0.010** |
| *Range maps-Field inventories* | 0.17 | -0.21 | 0.55 | 0.550 |
| *Museum records-Field inventories* | 0.35 | -0.03 | 0.73 | 0.080 |
| *Museum records-Range maps* | 0.18 | -0.17 | 0.53 | 0.450 |
| **PSV** |  |  |  |  |
| *Fine- vs Coarse-grained assemblages* | -0.01 | -0.04 | 0.01 | 0.260 |
| *Range maps-Field inventories* | 0.04 | 0.01 | 0.07 | **0.000** |
| *Museum records-Field inventories* | -0.03 | -0.07 | 0.00 | 0.050 |
| *Museum records-Range maps* | -0.08 | -0.11 | -0.05 | **0.000** |
| **PSC** |  |  |  |  |
| *Fine- vs Coarse-grained assemblages* | -0.04 | -0.06 | -0.01 | **0.000** |
| *Range maps-Field inventories* | 0.10 | 0.07 | 0.13 | **0.000** |
| *Museum records-Field inventories* | 0.05 | 0.01 | 0.08 | 0.054 |
| *Museum records-Range maps* | -0.05 | -0.09 | -0.02 | **0.000** |
