# Supplementary material for: Sensitivity of Metrics of Phylogenetic Structure to Scale, Source of Data and Species Pool of Hummingbird Assemblages along Elevational Gradients
Source: PLoS One. 2012 Apr 27;7(4):e35472. doi: 10.1371/journal.pone.0035472 (PMC3338702; doi:10.1371/journal.pone.0035472)
Supplement: Table S5 — Results of the ANCOVA analyses using the same number of samples per spatial grain. Significant P-values are highlighted in bold. (DOC) [file pone.0035472.s009.doc]

**Table S5. Results of the ANCOVA analyses using the same number of samples per spatial grain. Significant P-values are highlighted in bold.**

|  | **df** | **SS** | **MS** | ***F*** | ***P*** |
| --- | --- | --- | --- | --- | --- |
| *Species Richness* |  |  |  |  |  |
| Spatial grain | 1 | 2965.20 | 2965.20 | 85.45 | **0.00** |
| Data source | 2 | 8761.70 | 4380.90 | 126.25 | **0.00** |
| Elevation | 1 | 429.70 | 429.70 | 12.38 | **0.00** |
| *NRI* |  |  |  |  |  |
| Spatial grain | 1 | 0.67 | 0.67 | 0.32 | 0.57 |
| Data source | 2 | 8.83 | 4.41 | 2.12 | 0.13 |
| Elevation | 1 | 98.15 | 98.15 | 47.07 | **0.00** |
| *NTI* |  |  |  |  |  |
| Spatial grain | 1 | 6.69 | 6.69 | 6.06 | **0.01** |
| Data source | 2 | 5.69 | 2.84 | 2.58 | 0.08 |
| Elevation | 1 | 7.89 | 7.89 | 7.15 | **0.01** |
| *PSV* |  |  |  |  |  |
| Spatial grain | 1 | 0.01 | 0.01 | 1.67 | 0.20 |
| Data source | 2 | 0.03 | 0.02 | 3.17 | **0.05** |
| Elevation | 1 | 0.14 | 0.14 | 27.41 | **0.00** |
| *PSC* |  |  |  |  |  |
| Spatial grain | 1 | 0.21 | 0.21 | 25.25 | **0.00** |
| Data source | 2 | 0.10 | 0.05 | 6.04 | **0.00** |
| Elevation | 1 | 0.03 | 0.03 | 3.73 | **0.05** |
